# Hepatitis E Virus Detection in Swine Livers and Feces in Heilongjiang, Northeastern China

**DOI:** 10.3390/microorganisms13081899

**Published:** 2025-08-14

**Authors:** Haijuan He, Hai Li, Lei Yan, Gang Wang, Yonggang Liu, Tongqing An, Yabin Tu, Shujie Wang, Xuehui Cai

**Affiliations:** 1 State Key Laboratory for Animal Disease Control and Prevention, Harbin Veterinary Research Institute, Chinese Academy of Agriculture Science, Harbin 150069, China; he_haijuan@haas.cn (H.H.); yanlei316@126.com (L.Y.);; 2 Institute of Animal Husbandry, Heilongjiang Academy of Agriculture Sciences, Harbin 150086, China

**Keywords:** hepatitis E virus, genotype 3, genotype 4, infection, pig farm

## Abstract

Hepatitis E virus (HEV) is an emerging zoonotic pathogen capable of both human-to-human and animal-to-human transmission. However, limited data are available regarding HEV infections in pigs in Heilongjiang Province, China. To investigate the prevalence of HEV in pigs in this region, liver samples from diseased or deceased pigs and fecal samples from healthy pigs were collected and analyzed. A total of 82 liver samples and 86 fecal samples were obtained from 13 farms and tested for HEV genotypes 3 and 4 using nested RT-PCR assays targeting the *ORF2* gene. Samples with high viral loads were further subjected to direct sequencing and phylogenetic analysis. Overall, 32 samples tested positive for HEV RNA and were classified as genotype 3 or 4, with genotype 4 being the most prevalent. The identified subtypes included 3a, 4a, and 4d, among which subtype 4d was the most common. Phylogenetic analysis revealed that swine HEV genotype 3 (subtype 3a) and genotype 4 (subtypes 4a and 4d) clustered closely with reference sequences 3a/AB089824/JA10, 4a/JX9974449/NJ6, and 4d/JX997439/NJ5. These strains exhibited close genetic similarity to human HEV isolates previously reported in Tokyo, Japan, and eastern China. These findings indicate that HEV genotypes 3 and 4 are distributed in pig farms across Heilongjiang Province and suggest that zoonotic transmission between pigs and humans is frequent in China.

## 1. Introduction

Hepatitis E is a significant public health concern. Hepatitis E virus (HEV) infection is a global disease and the primary cause of both acute and chronic hepatitis in solid organ transplant recipients, with an estimated 20 million cases and 70,000 deaths reported annually worldwide [1,2,3,4]. HEV belongs to the genus *Herpesvirus* within the family Hepeviridae [5,6,7], which is a single-stranded, positive-sense, non-enveloped RNA virus with a size of 27–34 nm [8]. To date, eight closely related HEV genotypes (HEV-1 to HEV-8) have been identified. Among these, only four genotypes (HEV-1 to HEV-4) are known to cause infections in humans [9,10]. Genotypes 1 and 2 exclusively infect humans and are primarily transmitted through fecally contaminated drinking water in regions with inadequate sanitation. These genotypes are prevalent in developing countries, including those in Asia, Africa, and Central America [11]. In contrast, genotypes 3 and 4 have zoonotic potential, infecting both humans and animals, with pigs and wild boars identified as the primary hosts [12,13]. The first animal strain of HEV was isolated and characterized in domestic pigs in the United States in 1997 [14,15], marking the beginning of extensive research into swine as the main reservoir for HEV genotypes 3 and 4.

The HEV RNA genome is approximately 7.2 kb in length, with a 5′ cap structure and a 3′ poly (A) tail [16]. The genome consists of three open reading frames (ORFs). *ORF1* spans 5109 nucleotides and encodes a non-structural polyprotein of approximately 186 kDa composed of 1693 amino acids [17]. This polyprotein plays a crucial role in viral replication and infectivity and contains several functional domains, including methyltransferase, protease, RNA helicase, and RNA-dependent RNA polymerase [18,19]. These functional domains include methyltransferase, proteases, RNA helicases, and RNA-dependent RNA polymerases [20]. Therefore, *ORF1* is essential for the viral life cycle. *ORF2* is 1983 nucleotides long and encodes the viral capsid protein, which consists of 660 amino acids. This protein is responsible for viral particle assembly, interaction with host cells, and immunogenicity [21,22]. The P domain of the ORF2 protein serves as the primary antigenic region, inducing neutralizing antibodies, and is a key target for vaccine development [3]. Thus, *ORF2* plays a critical role in vaccine design and diagnostic applications. *ORF3* spans 372 nucleotides, with its 5′ end overlapping *ORF1* by four nucleotides and its 3′ end overlapping *ORF2* by 331 nucleotides. It encodes a small protein of 114 amino acids involved in viral particle morphogenesis and release [23].

Over the past three decades, the number of reported HEV cases has increased significantly in Asia. Human-to-human transmission is not considered an efficient mode of transmission. More effective transmission routes include consumption of contaminated water or food, or contact with infected animals [11,24]. In China, swine HEV was first detected in pig serum samples collected from central China in 2002 [25]. Since then, swine HEV has been recognized as a widespread zoonotic pathogen throughout the country. HEV genotypes 3 and 4 have been identified in swine samples from various regions of China, including eastern, southwestern, northeastern, northwestern, northern, and southern Provinces [26,27]. Swine are considered the primary reservoir for these genotypes, posing a significant risk of zoonotic transmission to humans. Despite this, there remains a limited amount of published data on the prevalence and genetic characteristics of HEV in certain regions.

The present study investigated the frequency of HEV infection in liver samples from diseased or deceased pigs and fecal samples from healthy pigs randomly collected from 66 farms in rural areas of eight different cities in Heilongjiang Province, northeastern China. The aim of this study is to provide information on the occurrence of HEV in pigs. Moreover, it seeks to study the genetic diversity of HEV and gain a deeper understanding of the phylogenetic relationship of the HEV strain that spreads between pigs and humans in China.

## 2. Material and Methods

### 2.1. Sample Collection and Ethical Considerations

Between 2013 and 2015, pig liver samples were randomly collected from diseased or deceased animals across 63 farms located in Harbin, Suihua, Daqing, Shuangyashan, Jiamusi, Yichun, and Mudanjiang in Heilongjiang Province, northeast China. A total of 82 pig samples were collected, including 43 from Harbin, 16 from Suihua, 7 from Daqing, 7 from Shuangyashan, 1 from Jiamusi, 5 from Yichun, and 3 from Mudanjiang. These samples were obtained from both diseased and deceased animals exhibiting various clinical symptoms and pathological lesions. Pigs exhibiting extreme lethargy were considered moribund and were humanely euthanized in accordance with humane protocols. In addition, fecal samples were randomly collected from 86 healthy pigs housed in three farms in Qiqihar, Heilongjiang Province, including 28 pigs from farm 1, 28 from farm 2, and 30 from farm 3. These pigs represented various age and physiological categories: nursery (7 pigs), growing (7 pigs), fattened (7 pigs in farm 1 and 2, 9 pigs in farm 3), and sows (7 pigs). All samples were immediately stored in ice boxes to maintain the cold chain during transportation to the laboratory, where liver samples were stored at −80 °C and fecal samples at −20 °C. The sample collection procedures were conducted in accordance with the Guide for the Care and Use of Laboratory Animals issued by the Ministry of Science and Technology of the People’s Republic of China. The study was approved by the Animal Ethics Committee of the Harbin Veterinary Research Institute, Chinese Academy of Agricultural Sciences (CAAS), under approval number SY-2013-SW-043 approved on 3 April 2013, and all procedures strictly followed the approved ethical guidelines.

### 2.2. Viral RNA Extraction

Prior to RNA extraction, liver samples were removed from the −80 °C freezer and thawed on ice. Approximately 15 mg of liver tissue was transferred into 300 μL of tissue lysis buffer and thoroughly homogenized using a tissue grinder. For fecal samples, approximately 15 mg of fecal material was suspended in 300 μL of tissue lysis buffer. Subsequently, 590 μL of RNase-free distilled water and 10 μL of proteinase K were added to each homogenate or fecal suspension. The mixture was vortexed thoroughly and incubated at 56 °C for 10 min. Following incubation, the samples were centrifuged at 12,000 rpm (approximately 13,400× *g*) for 5 min, and the supernatant was collected for HEV RNA extraction using the RNAprep Pure Tissue Kit (TIANGEN, Beijing, China), following the manufacturer’s instructions. Liver tissue from specific pathogen free (SPF) pigs previously stored in the laboratory was used as a negative control.

### 2.3. Reverse Transcription Polymerase Chain Reaction (RT-PCR)/Nested PCR

Following RNA extraction, RT-PCR with a nested amplification strategy was conducted using two sets of HEV-specific primers targeting the *ORF2* region. The amplification yielded a 436 bp fragment for genotype 3 and a 270 bp fragment for genotype 4. The outer nested primers used were 5′-GACAGAATTRATTTCGTCGG-3′ and 5′-AADGTYTTRGARTACTGCTG-3′, while the inner nested primers were 5′-GTCTCRGCCAATGGCGAGCCGAC-3′ and 5′-ACNGTRTCNGARACATACAT-3′. Sterile distilled water was included as a negative control to monitor potential contamination. Both the first round and second round PCR amplifications were performed under the following thermal cycling conditions: initial denaturation at 95 °C for 5 min, followed by 30 cycles of denaturation at 94 °C for 1 min, annealing at 55 °C for 1 min, extension at 72 °C for 30 s, and a final extension at 72 °C for 10 min. Swine HEV isolates 3a (swHLJ-HRBJCL/2015/CHN) and 4d (swHLJ-BXBX/2015/CHN) were selected for extended *ORF2* sequencing using previously published primer sets [28,29]. The amplified PCR products were resolved on a 1.2% agarose gel and visualized using SYBR^®^ Safe staining (Life Technologies, Austin, TX, USA).

### 2.4. Sequencing and Phylogenetic Analysis

PCR products corresponding to partial *ORF2* genomic regions were separated on a 1.2% agarose gel, excised, and purified using the EasyPure Gel Extraction Kit (TransGen, Beijing, China). Direct sequencing was carried out using the dideoxynucleotide chain termination method with the ABI PRISM BigDye Terminator Cycle Sequencing Ready Reaction Kit v1 and the ABI PRISM 3730 Genetic Analyzer (Applied Biosystems, Foster City, CA, USA). Sequence similarity between the *ORF2* nucleotide sequences obtained from HEV-positive samples and reference HEV *ORF2* sequences available in GenBank was assessed using the BLAST online search tool (http://blast.ncbi.nlm.nih.gov (accessed on 15 October 2015)). Multiple sequence alignment of the *ORF2* coding regions of representative HEV strains was performed using MUSCLE 3.8.31 [30]. An unrooted neighbor-joining phylogenetic tree was constructed based on the aligned partial *ORF2* sequences and selected HEV subtype reference strains from GenBank (Table 1) using MEGA V 7.0 software [31]. Evolutionary distances were calculated using the maximum composite likelihood method, with statistical support for tree topology assessed via 1000 bootstrap replicates. Branches supported by less than 50% bootstrap values were collapsed and indicated above the respective branches.

### 2.5. Nucleotide Sequence Accession Numbers

The partial *ORF2* sequences derived from the HEV-positive samples analyzed in this study have been deposited in GenBank under the accession numbers KU130364 to KU130395, as detailed in Table 2.

## 3. Results

### 3.1. Prevalence of HEV in Pigs on Farms

RNA of HEV was detected using nested RT-PCR with both sets of primers, and the amplified products were visualized on a 1.2% agarose gel. As shown in Table 3 and Table 4, among the 168 samples collected from pigs, a total of 32 samples tested positive for HEV. The resulting fragments were purified and subjected to direct sequencing, and the sequences were subsequently analyzed using the BLAST database on NCBI. The BLAST results indicated that 32 sequences originating from 21 pig farms across seven districts were identified as partial *ORF2* sequences of HEV. Thus, the overall HEV infection proportion among the 168 collected samples was 19.1% (32/168). Among the 82 liver samples collected from diseased or deceased pigs, 27 samples tested positive (Table 2), yielding an HEV infection proportion of 32.9% (27/82). As shown in Table 3, among the 86 fecal samples collected from healthy pigs in three different farms in Qiqihar City, Heilongjiang Province, 5 samples tested positive: 1 sample was positive among 28 pigs in Farm 1, 2 samples were positive among 28 pigs in Farm 2, and 2 samples were positive among 30 pigs in Farm 3, resulting in an HEV infection proportion of 5.8% (5/86). All five positive samples were obtained from fattening pigs.

### 3.2. Genotypes of HEV in Pigs on Farms in Heilongjiang Province

**Figure 1:**
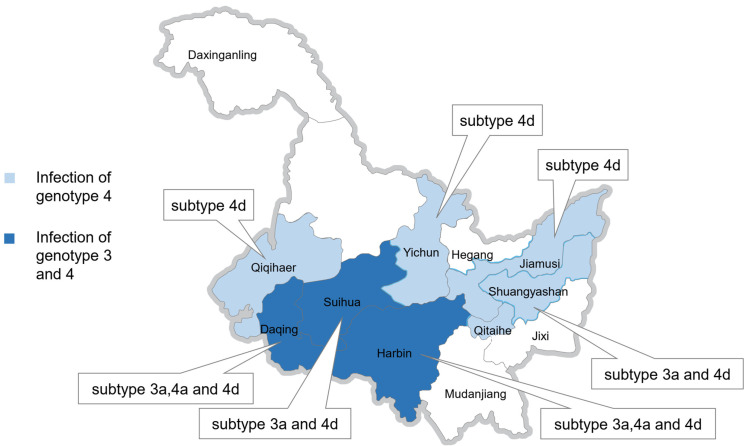
Distribution of confirmed genotypes and subtypes of swine HEV detected from seven districts in Heilongjiang Province of China.

Sequence analysis of HEV-infected pigs revealed that 26 sequences originating from 21 pig farms across seven districts (Harbin, Suihua, Daqing, Shuangyashan, Jiamusi, Yichun, and Mudanjiang) were classified as genotype 4, while the remaining 6 sequences were classified as genotype 3. In healthy pigs, sequence analysis revealed that five sequences originating from three pig farms in Qiqihar were also classified as genotype 4. These findings indicate that genotype 4 is the predominant genotype among pigs in Heilongjiang Province, northeastern China (Table 3 and Table 4, and Figure 1).

### 3.3. Subtypes of HEV Genotypes 3 and 4 on Farms in Heilongjiang Province

**Figure 2:**
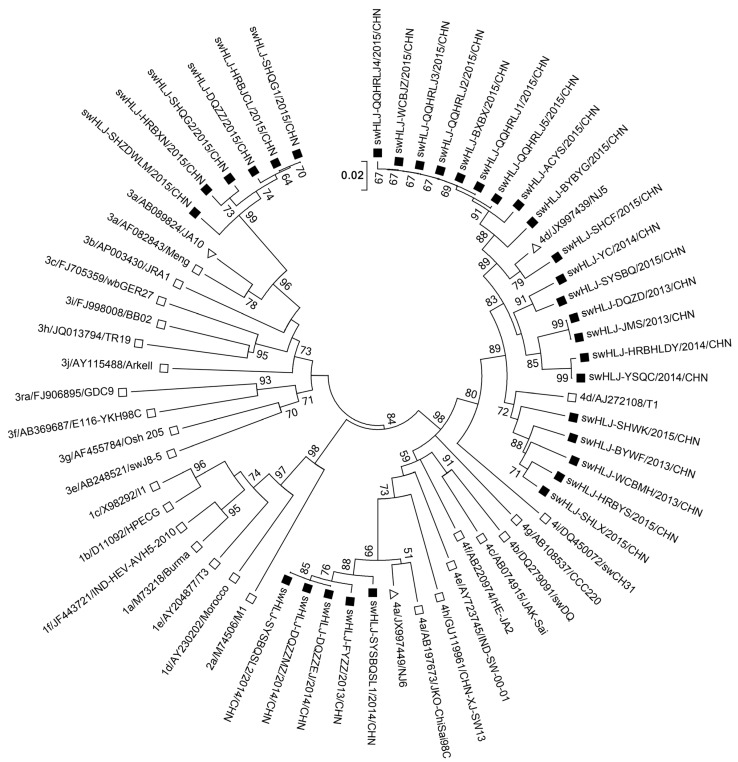
A phylogenetic tree of HEV isolates in Heilongjiang, northeastern China. The phylogenetic tree was produced with a 251 bp *ORF2* sequence (the positions of the 5’and 3’end of the *ORF2* sequence was 6397 and 6647; the corresponding accession number of the sequence used to count the positions was AF082843, which was the first swine HEV isolated by Meng in 1997) alignment of 32 strains from this study and 29 other reference sequences using the neighbor-joining method. The tree was evaluated by using the interior branch test method with MEGA7 software. The percentage of bootstrap support is shown by values at the branch nodes of the tree. Subtype, GenBank accession number, and strain are indicated in referenced HEV isolates. Only partial branches that were sufficient for elucidating the relationship between the study strains and their related strains are shown. Black squares represent the strains identified in this study; white squares denote the typical HEV genotypes and subtypes of GenBank sequences according to Smith et al. [33]; white triangles denote the GenBank sequences with the highest sequence homology to our typical sequences; and subtype 3ra is derived from the rabbit. The scale bar indicates the nucleotide substitutions per position.

Figure 2 presents the phylogenetic tree constructed from the 32 isolates obtained in this study and their closest matching sequences in GenBank. To further classify the subtypes of HEV genotypes 3 and 4, we included additional well-characterized HEV strains from other regions worldwide [27,32]. Phylogenetic analysis demonstrated that HEV genotype 4 could be divided into two subtypes: 5 of the 26 genotype 4 sequences were classified as subtype 4a, sharing >98.2% nucleotide identity by pairwise comparison, while 21 of the 26 sequences were classified as subtype 4d, with 89.3–100% nucleotide identity (Table 3 and Table 4 and Table S1). All six genotype 3 sequences were classified as subtype 3a, sharing 95.6–100% nucleotide identity and exhibiting the closest relationship (90.4–93.2% nucleotide identity) with a human-derived HEV subtype 3a sequence (ID: AB089824) from Tokyo, Japan (Table S1).

### 3.4. Phylogenetic Analysis of HEV ORF2 Sequence

**Figure 3:**
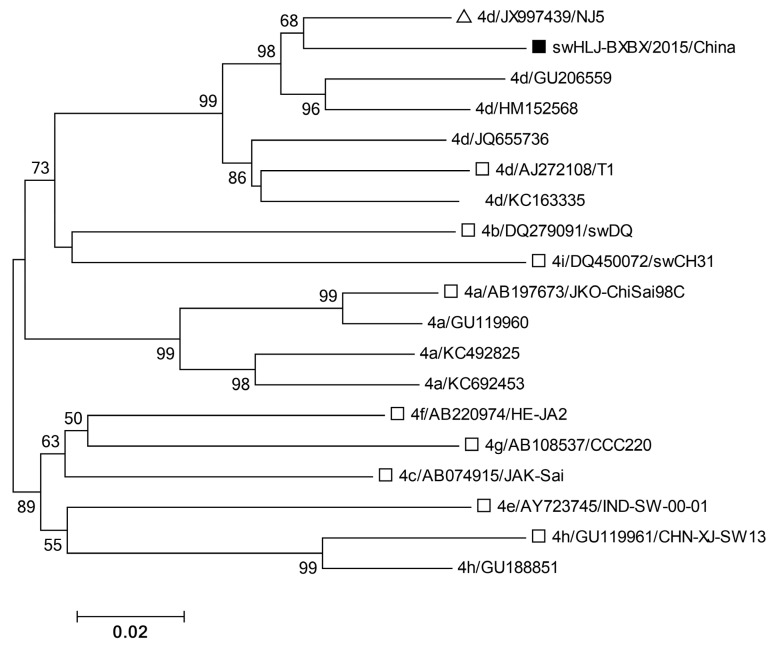
A phylogenetic tree demonstrating the alignment of the partial *ORF2* 852 bp of the swHLJ-BXBX/2015/CHN strain from the present study. The referenced genotype 4 isolates with the partial *ORF2* 852 bp available in GenBank were also included (except for the referenced JX997439 with 666bp within the partial *ORF2* 852 bp). The tree was constructed by using the neighbor-joining method and evaluated by using the interior branch test method with MEGA7 software. The percentage of bootstrap support is indicated at each node. Subtype, source, GenBank accession number, and country of origin are indicated in the referenced HEV isolates. Strains identified in this study are indicated by black squares and the highest sequence homology to our typical sequences are indicated by white triangles. White squares denote the typical HEV genotypes and subtypes of GenBank sequences according to Smith et al. [33]. Bootstrap values were determined with 1000 replicates. Scale bars indicate the nucleotide substitutions per site.

**Figure 4:**
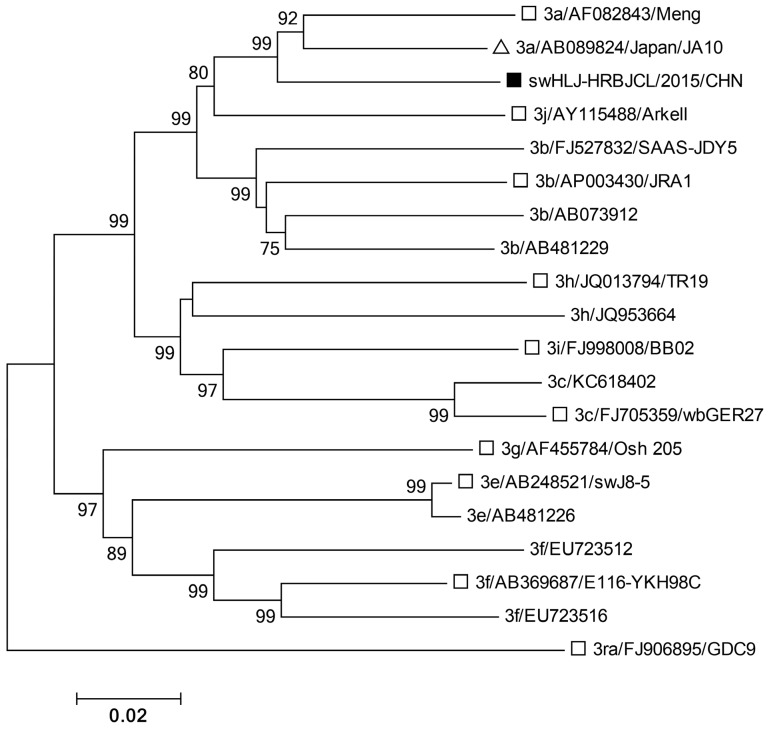
A phylogenetic tree demonstrating the alignment of the partial *ORF2* 852 bp of the swHLJ-HRBJCL/2015/CHN strain from the present study. The complete *ORF2* sequence of swHLJ-HRBJCL/2015/CHN and referenced genotype 3 isolates with the complete *ORF2* sequence available in GenBank (except for the referenced HM439285 with 1681 bp within the complete *ORF2* sequence). The tree was constructed by using the neighbor-joining method and evaluated by using the interior branch test method with MEGA7 software. The percentage of bootstrap support is indicated at each node. Subtype, source, GenBank accession number, and country of origin are indicated in the referenced HEV isolates. Strains identified in this study are indicated by black squares and the highest sequence homology to our typical sequences are indicated by white triangles. White squares denote the typical HEV genotypes and subtypes of GenBank sequences according to Smith et al. [33]. Bootstrap values were determined with 1000 replicates. Scale bars indicate the nucleotide substitutions per site.

Five genotype 4 isolates in the 4a group exhibited greater than 98.2% sequence identity (Table S1) and clustered closely with a human strain (GenBank accession no. JX997449 [NJ6]) isolated in eastern China [34] (Figure 2). Similarly, 21 genotype 4d isolates shared 89.3–100% sequence identity and clustered closely with a human strain (JX997439 [NJ5]), also isolated in eastern China [34] (Figure 2). Due to the quality and quantity of liver samples (some from deceased pigs), the partial *ORF2* sequence (852 bp) of subtype 4d (swHLJ-BXBX/2015/CHN) and the complete *ORF2* sequence of subtype 3a (swHLJ-HRBJCL/2015/CHN) were successfully obtained. Furthermore, the partial ORF2 sequence (852 bp) of subtype 4d, swHLJ-BXBX/2015/CHN (Accession: KU130377), was found to cluster closely with the human strain JX997439/NJ5 (Figure 3), exhibiting 93.7% sequence homology (Table S1). These results suggest that HEV subtypes 4a and 4d may be involved in cross species transmission and have likely spread throughout Heilongjiang Province and eastern China. A BLAST search and phylogenetic analysis based on the complete *ORF2* sequence of swHLJ-HRBJCL/2015/CHN (Accession: KU130391) and other available genotype 3 strains in GenBank confirmed that the swHLJ-HRBJCL/2015/CHN strain belongs to genotype 3a and clusters closely with a human isolate from Japan (JA10; AB089824) [35] (Figure 4), with 92.2% sequence homology (Table S1). Additionally, the complete *ORF2* sequence homology between swHLJ-HRBJCL/2015/CHN and the first isolated genotype 3b FJ527832 (SAAS-JDY5) from swine in China was 89.2% (Figure 4 and Table S1). These findings indicate that this genotype 3 strain is more closely related to human isolates from Japan than to other swine strains from China (Figure 4).

## 4. Discussion

HEV is the fifth known etiological agent of viral hepatitis in humans and is considered the most prevalent cause of acute hepatitis and jaundice globally [36]. Initially identified as non-A, non-B hepatitis in the 1980s, HEV has become widespread, particularly in developing countries [37]. Transmission occurs both via the fecal–oral route among humans and from animals to humans through fecal contamination, direct contact, or consumption of contaminated meat products. HEV has been detected in pig herds, pork products, and environmental samples, raising concerns about its zoonotic potential [38,39]. Genotypes 3 and 4 of HEV are recognized as zoonotic and have been associated with sporadic cases of hepatitis E in Europe and east Asia, where they infect various mammalian hosts, including pigs, deer, rabbits, and humans [40,41]. In northeastern China, data on the prevalence of HEV infection in swine are limited. This survey aimed to assess the prevalence of HEV genotypes 3 and 4 in both healthy and diseased pigs, a population potentially at higher risk for chronic HEV infection. Given the close human–swine interactions on pig farms, the risk of zoonotic transmission is likely elevated. The prevalence of HEV in liver samples from sick or deceased pigs (32.9%, 27/82) was significantly higher than in fecal samples from healthy pigs (5.8%, 5/86), suggesting that HEV strains circulating in swine in northeastern China may have more severe pathogenic effects in immunocompromised or diseased pigs.

The HEV genome exhibits high genetic diversity, particularly among genotypes 3 and 4, which display considerable genetic heterogeneity and are further classified into multiple subtypes. Subtype classification requires at least three complete, phylogenetically and epidemiologically distinct viral genomes. Due to these stringent criteria, many HEV strains remain unclassified [42]. To date, genotype 3 comprises ten subtypes (3a–3j). In this study, the predominant subtype identified was 3a. Since 2006, genotype 3 has been detected in swine in central and southwestern China, as well as in both humans and swine in eastern China [43,44]. Previous studies have reported genotype 3 infections in humans in eastern China, with sequence homology ranging from 96.4% to 97.9% compared to swine strains, suggesting a possible zoonotic origin from local swine populations [45]. This study reports, for the first time, the identification of HEV genotype 3 subtype 3a in Heilongjiang Province, China. The high sequence similarity between the swHLJ-HRBJCL/2015/CHN strain and a human isolate from Japan (AB089824) suggests that the HEV genotype 3a strains circulating in swine in Heilongjiang may have originated from Japan. This finding indicates that genotype 3a strains may represent an emerging threat to both swine and potentially humans in this region.

HEV infection in pregnant women can lead to serious complications, including premature birth, stillbirth, and vertical transmission to the fetus. HEV genotype 4 is the predominant cause of hepatitis E in China and has been shown to replicate in placental tissue, causing severe histopathological damage and vertical transmission [46]. Moreover, human genotype 4 strains exhibit over 90% sequence homology with swine strains nationwide [27,34]. Currently, genotype 4 includes seven subtypes (4a–4g). In this study, the primary subtypes identified were 4a and 4d, representing the first reported subtypes in Heilongjiang Province. A BLAST search in GenBank revealed that some genotype 4 isolates obtained in this study exhibited the highest sequence homology with the human strain NJ5 (JX997439) previously identified in Jiangsu Province, China. These findings suggest that these isolates may not be indigenous to Heilongjiang and could be involved in cross species transmission between swine and humans within China. Furthermore, among the seven HEV-positive regions tested, four showed the presence of multiple subtypes, indicating co-infection with different HEV subtypes in the same geographic area. Of the 32 HEV-positive samples, 27 were obtained from diseased or deceased pigs. The variability in clinical symptoms and histopathological lesions observed suggests that these pigs were likely co-infected with other pathogens in addition to HEV.

In conclusion, a total of 168 samples were collected from eight regions in Heilongjiang Province, northeastern China, including 82 samples from diseased or deceased pigs and 86 from healthy pigs. Of these, 32 tested positive for HEV, with genotypes identified as type 3 and type 4, and subtypes classified as 3a, 4a, and 4d. Phylogenetic analysis revealed a close genetic relationship between these isolates and human strains from China and Japan, underscoring the zoonotic potential and the need for continued surveillance.

## Figures and Tables

**Table 1:** Reference known HEV subtype strains in GenBank.

Accession	Strain	Host	Genotype	Subtype
AB089824	3a/AB089824/JA10	Homo sapiens	3	3a
AF082843	3a/AF082843/Meng	Swine	3	3a
AP003430	3b/AP003430/JRA1	Homo sapiens	3	3b
FJ705359	3c/FJ705359/wbGER27	Wild boar	3	3c
FJ998008	3i/FJ998008/BB02	Wild boar	3	3i
JQ013794	3h/JQ013794/TR19	Homo sapiens	3	3h
AY115488	3j/AY115488/ArKell	Swine	3	3j
FJ906895	3ra/FJ906895/GDC9	Rabbit	3	3ra
AB369687	3f/AB369687/E116-YKH98C	Homo sapiens	3	3f
AF455784	3g/AF455784/Osh 205	Swine	3	3g
AB248521	3e/AB248521/swJ8-5	Swine	3	3e
X98292	1c/X98292/I1	Homo sapiens	1	1c
D11092	1b/D11092/HPECG	Homo sapiens	1	1b
JF443721	1f/JF443721/IND-HEV-AVH5-2010	Homo sapiens	1	1f
M73218	1a/M73218/Burma	Homo sapiens	1	1a
AY204877	1e/AY204877/T3	Homo sapiens	1	1e
AY230202	1d/AY230202/Morocco	Homo sapiens	1	1d
M74506	2a/M74506/M1	Homo sapiens	2	2a
JX997449	4a/JX997449/NJ6	Homo sapiens	4	4a
AB197673	4a/AB197673/JKO-ChiSai98C	Homo sapiens	4	4a
GU119961	4h/GU119961/CHN-XJ-SW13	Swine	4	4a
AY723745	4e/AY723745/IND-SW-00-01	Swine	4	4e
AB220974	4f/AB220974/HE-JA2	Homo sapiens	4	4f
AB074915	4c/AB074915/JAK-Sai	Homo sapiens	4	4c
DQ279091	4b/DQ279091/swDQ	Swine	4	4b
AB108537	4g/AB108537/CCC220	Homo sapiens	4	4g
DQ450072	4i/DQ450072/swCH31	Swine	4	4i
AJ272108	4d/AJ272108/T1	Homo sapiens	4	4d
JX997439	4d/JX997439/NJ5	Homo sapiens	4	4d

**Table 2:** 32 samples identified as positive for HEV in this study.

Accession	Strain	Isolation Source	Genotype:	Subtype
KU130364	swHLJ-FYZZ/2013/CHN	Liver	4	4a
KU130365	swHLJ-DQZZEJ/2014/CHN	Liver	4	4a
KU130366	swHLJ-DQZZMZ/2014/CHN	Liver	4	4a
KU130367	swHLJ-SYSBQSL1/2014/CHN	Liver	4	4a
KU130368	swHLJ-SYSBQSL2/2014/CHN	Liver	4	4a
KU130369	swHLJ-BYWF/2013/CHN	Liver	4	4d
KU130370	swHLJ-DQZD/2013/CHN	Liver	4	4d
KU130371	swHLJ-JMS/2013/CHN	Liver	4	4d
KU130372	swHLJ-WCBMH/2013/CHN	Liver	4	4d
KU130373	swHLJ-HRBHLDY/2014/CHN	Liver	4	4d
KU130374	swHLJ-YC/2014/CHN	Liver	4	4d
KU130375	swHLJ-YSQC/2014/CHN	Liver	4	4d
KU130376	swHLJ-ACYS/2015/CHN	Liver	4	4d
KU130377	swHLJ-BXBX/2015/CHN	Liver	4	4d
KU130378	swHLJ-BYBYG/2015/CHN	Liver	4	4d
KU130379	swHLJ-HRBYS/2015/CHN	Liver	4	4d
KU130380	swHLJ-QQHRLJ1/2015/CHN	Fecal sample	4	4d
KU130381	swHLJ-QQHRLJ2/2015/CHN	Fecal sample	4	4d
KU130382	swHLJ-QQHRLJ3/2015/CHN	Fecal sample	4	4d
KU130383	swHLJ-QQHRLJ4/2015/CHN	Fecal sample	4	4d
KU130384	swHLJ-QQHRLJ5/2015/CHN	Fecal sample	4	4d
KU130385	swHLJ-SHCF/2015/CHN	Liver	4	4d
KU130386	swHLJ-SHLX/2015/CHN	Liver	4	4d
KU130387	swHLJ-SHWK/2015/CHN	Liver	4	4d
KU130388	swHLJ-SYSBQ/2015/CHN	Liver	4	4d
KU130389	swHLJ-WCBJZ/2015/CHN	Liver	4	4d
KU130390	swHLJ-DQZZ/2015/CHN	Liver	3	3a
KU130391	swHLJ-HRBJCL/2015/CHN	Liver	3	3a
KU130392	swHLJ-HRBXN/2015/CHN	Liver	3	3a
KU130393	swHLJ-SHQG1/2015/CHN	Liver	3	3a
KU130394	swHLJ-SHQG2/2015/CHN	Liver	3	3a
KU130395	swHLJ-SHZDWLM/2015/CHN	Liver	3	3a

**Table 3:** Detection and characterization of hepatitis E virus in 82 samples from 7 districts of Heilongjiang Province in this study.

Number of Samples (*n* = 82)	Number of Pig Farms (*n* = 63)	No. Positive/No. Tested (%)	HEV Subtypes	Location (city)	Source
		27/82(32.9)			
43	30	12/43(27.9)	3a, 4a, 4d	Harbin	Diseased/deceased pigs, Liver samples
16	10	6/16(37.5)	3a, 4d	Suihua	Diseased/deceased pigs, Liver samples
7	7	4/7(57.1)	3a, 4a, 4d	Daqing	Diseased/deceased pigs, Liver samples
7	7	3/7(42.8)	4a, 4d	Shuangyashan	Diseased/deceased pigs, Liver samples
1	1	1/1(100)	4d	Jiamusi	Diseased/deceased pigs, Liver samples
5	5	1/5(20)	4d	Yichun	Diseased/deceased pigs, Liver samples
3	3	0/3(0)	--	Mudanjiang	Diseased/deceased pigs, Liver samples

**Table 4:** Detection and characterization of hepatitis E virus in 86 samples from 3 farms in Qiqihar of Heilongjiang Province in this study.

Number of Samples (*n* = 86)	Name of Pig Farms (*n* = 3)	No. Positive/No. Tested (%)	HEV Subtypes	Location (city)	Source
		5/86(5.8)			
28	Farm 1	1/28(3.5)	4d	Qiqihar	Healthy pigs, Fecal samples
28	Farm 2	2/28(7.1)	4d	Qiqihar	Healthy pigs, Fecal samples
30	Farm 3	2/30(6.7)	4d	Qiqihar	Healthy pigs, Fecal samples

## Data Availability

The original contributions presented in this study are included in the article/Supplementary Material. Further inquiries can be directed to the corresponding authors.

## Supplementary Materials

The following supporting information can be downloaded at: https://www.mdpi.com/article/10.3390/microorganisms13081899/s1, Table S1: Pairwise comparison of HEV *ORF2* sequences using BLASTN.
